# Heat Build-Up and Rolling Resistance Analysis of a Solid Tire: Experimental Observation and Numerical Simulation with Thermo-Mechanical Coupling Method

**DOI:** 10.3390/polym14112210

**Published:** 2022-05-30

**Authors:** Hong He, Jinming Liu, Yaru Zhang, Xue Han, William V. Mars, Liqun Zhang, Fanzhu Li

**Affiliations:** 1 State Key Laboratory of Organic-Inorganic Composites, Beijing University of Chemical Technology, Beijing 100029, China; hehong@mail.buct.edu.cn (H.H.); heykeiu@163.com (J.L.); 18811610836@163.com (Y.Z.); zhanglq@mail.buct.edu.cn (L.Z.); 2 College of Mechanical and Electrical Engineering, Beijing University of Chemical Technology, Beijing 100029, China; 3 Key Laboratory of Beijing City on Preparation and Processing of Novel Polymer Materials, Beijing University of Chemical Technology, Beijing 100029, China; 2018020389@mail.buct.edu.cn; 4 Endurica LLC, 1219 West Main Cross St., Suite 201, Findlay, OH 45840, USA; wvmars@endurica.com

**Keywords:** rolling resistance, heat build-up, thermo-mechanical, viscoelastic, Lagrangian–Eulerian model

## Abstract

The hysteresis of rubber materials due to deformation and viscoelasticity is the main reason for the heat build-up (HBU) and rolling resistance (RR) of the rolling tire. It is important to realize the high precision prediction of HBU and RR of tire for the optimal design of high-performance fuel-saving tire. In this work, a thermo-mechanical coupling method based on Endurica and Abaqus co-simulation was used to predict the steady-state temperature distribution and RR of three finite element models (Lagrangian–Eulerian model, Lagrangian model, and Plane Strain model) of the solid tires under different loads and rotating speeds. The simulation results were compared with the experimental results. The Kraus self-heating model was utilized in the thermo-mechanical coupling method, which realized the quantitative relationship between the dynamic loss modulus of rubber and the loading conditions (temperature, strain, and strain rate). Special attention was paid to the determination of the material parameters in the Kraus self-heating model. The comparison between simulation results and experimental results shows that the Lagrangian model had the highest prediction accuracy, and the average prediction errors of the steady-state surface temperature and RR under three loading conditions were 3.4% and 7.9%, respectively. The Lagrangian–Eulerian model came in the second with average errors of 9.7% and 11.1%, respectively. The Plane Strain model had the worst prediction accuracy, with the average errors of 21.4% and 44.6%, respectively. In terms of the simulation time, the Plane Strain model had the lowest cost, and the average calculation time was 1143 s. The Lagrangian–Eulerian model took the second place, with an average calculation time of 2621 s. The Lagrangian model had the highest computation cost, with an average time of 5597 s. The comparison between the simulation results and the experimental results verified the effectiveness of the thermo-mechanical coupling analysis method. The methods of three finite element models of the solid tires in this work can provide some reference for the optimization design of elastomeric components (Lagrangian model), pneumatic tires (Lagrangian–Eulerian model), and non-pneumatic tires (Plane Strain model).

## 1. Introduction

Rolling resistance (RR) is a key performance index in the tire industry, which is closely related to the durability and energy consumption of tires [1,2,3]. Both the RR and heat build-up (HBU) of the tire are derived from the viscoelastic effect of the rubber material [4,5,6]. With the development of computer power and theoretical algorithms, the finite element method (FEM) or finite element analysis (FEA) has become an efficient, popular, and practical numerical analysis method [7,8]. Therefore, using FEM to construct high-precision tire RR prediction is of great significance to the preliminary design and cost control of fuel-saving and durable tires [9].

According to the search in WEB of Science, a considerable amount of scientific literature on thermo-mechanical analysis of the tire RR has been steadily accumulating, producing over 800 publications so far. Some outstanding works have been done in this field. For example, the pioneering work in 1981 by Whicker et al. [10] proposed the conceptual outline of an interactive, combined thermo-mechanical model containing three major modules: one to handle tire deformations, one to handle heat generation within the tire, and one to handle heat transfer within the tire and to the environment. The modules interact through feedback loops between them. Shida et al. [11] proposed a practical RR simulation method for tires using a static FEM and the method computed hysteresis loops by introducing a viscoelastic phase lag between the stress and strain profiles. The steady state RR simulation of a passenger radial tire using this approach accurately captures key trends: the effect of cord angle on loss, also the effects of tire size and geometry variations, inflation pressure, and load. Using a hyper-viscoelastic finite element model, Veeramurthy et al. [12] determined the thickness of spokes, the shear band thickness, and shear modulus of polyurethane (PU) on the overall performance of non-pneumatic tires through design of experiments (DOE) and sensitivity analysis. The results show that the shear modulus and shear band thickness of PU have the greatest influence on rolling resistance, and higher shear modulus and shear band thickness will lead to lower RR. Yoo et al. [13] developed a numerical tool that quantifies non-inflatable tire rolling energy loss and corresponding internal heat build-up for hexagonal honeycomb spokes. The developed tool combining the viscoelastic material model with the aerodynamic heat loss quantifies well the hysteretic energy loss and the temperature distribution at each component of the non-pneumatic tire. Nyaaba et al. [14] performed a sequentially coupled thermo-mechanical rolling analysis of an ultra-large mining dump truck tire. The Abaqus software was used to provide stress, strain, and temperature data for the computation of the tire’s component fatigue performance in the rubber fatigue solver Endurica CL. The results showed that the belt endings, lower sidewall, and tread lug corners are susceptible to crack initiation and subsequent failure. Li et al. [15] predicted the transient temperature and RR of a solid rubber tire based on an established thermo-mechanical coupling approach and nonlinear viscoelastic theory by using FEM. The dependence of RR and heat build-up on thermal conductivity and loss factor of rubber materials were investigated by parametric numerical experiments. By using Prony series and parallel rheological framework (PRF) models, Aldhufairi et al. [16] studied the role of linear and nonlinear viscoelastic representation of materials to verify the calculation accuracy of tire RR. With the combination of the improved convergence at high speed of tires in Abaqus and the incremental, critical plane fatigue analysis procedures of Endurica DT, a pioneering work was done by Mars et al. [17]. They studied the standing wave development, self-heating, and fatigue during regulatory high-speed tire testing protocols. In their work, the strain amplitude and temperature dependence of the viscoelastic loss modulus were specified via the Kraus self-heating model [18] in the heat build-up calculation.

A lot of groundbreaking work has been reported; however, most studies lack systematic and comprehensive material characterization methods to establish reliable material parameters in the material models such as hyperelastic constitutive model, self-heating model, or lack sufficient experimental validation and verification. On the other hand, many reports have focused on carbon black-filled natural rubber systems rather than silica-reinforced solution styrene-butadiene rubber (S−SBR) systems, which are mostly used in passenger car green tires. In addition, three types of tire finite element model are often adopted [19,20], including: (1) Lagrangian–Eulerian finite element model, which is widely used in the simulation analysis of pneumatic tire performance. For example, Korunović et al. [21] established a Lagrangian–Eulerian finite element tire model and verified the validity of the model by comparing the simulation results with the experimental results. Nackenhorst et al. [22] conducted thermo-mechanical coupling analysis of tire model under complex road surface based on the finite element modeling method of Arbitrary Lagrangian Eulerianian (ALE) formula. Futamura et al. [23] proposed a deformation index to calculate the energy loss of tire. The deformation index concept was applied to the thermo-mechanical coupling analysis, where it simplified the fully coupled iterative FEA method into a noniterative computational method. (2) Lagrangian finite element model is often used in the simulation analysis of elastomeric components such as tire, bushing, bearing, and seal, etc. Grujicic et al. [24] carried out a series of transient, non-linear dynamics finite element analyses to investigate the interactions between a stereotypical pneumatic tire and sand during off-road vehicle travel within ABAQUS/Explicit, and the Lagrangian approach is used. Mars et al. [25] established a Lagrangian finite element model of backer pad used in Abrams tank track system. The backer pad design was digitally twinned to show how complex in-service conditions can be evaluated virtually. The digital twin of the backer pad yielded failure modes quite similar to those observed in experiments, and gave realistic estimates of operating temperature and fatigue life. Rugsaj et al. [26] developed a Lagrangian finite element model of non-pneumatic tire that can be used to evaluate the performance and mechanical behaviors of the tire during usage and testing and to help designing the non-pneumatic tire with the different geometries and materials. (3) Plane strain finite element model is widely used in practice where the stress state varies slowly with depth in a thick component of constant cross section. The method saves computation time by reducing the number of degrees of freedom in the model. Ju et al. [27] designed two hexagonal honeycomb spoke structures for non-pneumatic tires by using 2D plane strain model. Researchers from Clemson University [28,29,30,31] used the plane strain finite element model to study the effects of geometric parameters and material properties of the non-pneumatic tire on the tire performances such as static load-deflection, vibration at high-speed rolling, and energy loss from impact rolling over obstacles. All the above three models can be used for optimal design and performance prediction of rubber products, but little work has been done to investigate their differences in self-heating and rolling resistance prediction of tires.

The purpose of this work is to establish a combined Abaqus and Endurica thermo-mechanical coupling simulation method that can predict the self-heating of rubber and the rolling resistance of tires. The feasibility and reliability of the method were verified by three finite element models of a small solid rubber tire made of silica reinforced S−SBR under different rolling conditions. To gain a clearer picture, this article is organized as follows. First, the experiments used to calibrate the material parameters and validate the simulation results were introduced. Second, the workflow of thermo-mechanical coupling method used in this work was outlined. Special attention was paid to the determination of the material parameters in the hyperelastic model and of the Kraus self-heating model. Moreover, a simple eight-node unit cell model was used as a reference case to calculate the key parameter—scaling factor. Then, three finite element models of solid tire were established, and the HBU and RR under the boundary conditions of different load and rolling velocity were calculated by using the established thermo-mechanical coupling method. The finite element calculation time of these three solid tire models was also calculated. The simulation results were compared with the experimental results.

## 2. Experiment

### 2.1. Material Preparation

The rubber material formula of the green solid tire is shown in Table 1. Based on the material formula, the S−SBR rubber mixing process is: First, the raw S−SBR rubber was masticated in an open mill. Zinc oxide, stearic acid, and antioxidant were added in sequence and mixed, then silica was added and mixed. Finally, the accelerator and sulfur were added and mixed to obtain the final rubber compound.

S−SBR2550 is purchased from Korea LG Chemical Co. Ltd. (Seoul, Korea), VN3 is from Germany Evonik Industrial Group (Qingdao) Co., Ltd. (Qingdao, China), Si69 is from Nanjing Dawning Chemical Co., Ltd. (Nanjing, China), and ZnO, SA, S, CBS, D, and RD are all commercially available. The rubber material, filler and other additives were masticated in an open mill of Guangdong Zhanjiang Rubber and Plastic Machinery Factory. The optimum curing time (t_90_) of the S−SBR compound was measured by the curemeter of Beijing Huanfeng Machinery Factory.

### 2.2. Solid Rubber Tire and Specimen Preparation

**Figure 1:**
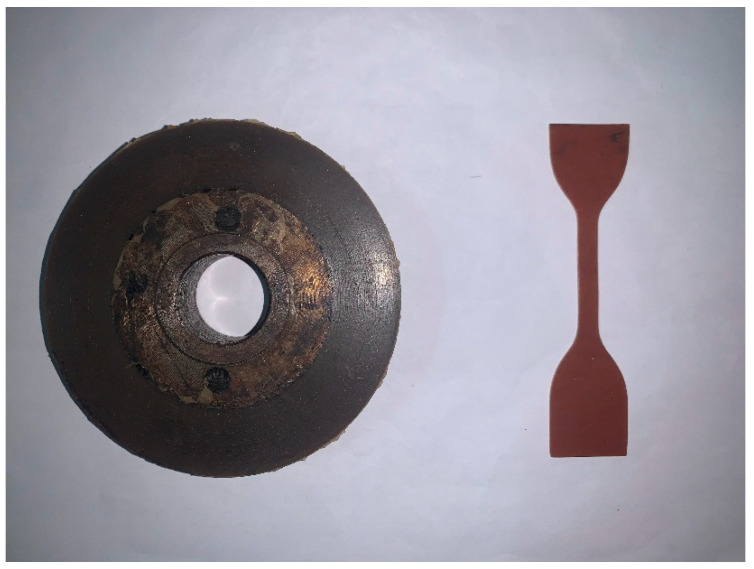
Solid rubber tires (**left**) and dumbbell rubber specimen (**right**).

The S−SBR compound was filled into a solid tire mold for high temperature vulcanization, and was then vulcanized at 150 °C and 15 MPa in a curing press. The final molded dimensions of the solid rubber tire were: radius of 50 mm, thickness of 20 mm, and width of 18 mm. Several dumbbell rubber specimens were prepared by the same vulcanization method and used for the subsequent tensile recovery test. Figure 1 shows a solid rubber tire and a dumbbell rubber specimen.

### 2.3. Thermal Test

A Hot Disk TPS1500 by Hot Disk AB of Sweden was used to conduct thermal tests on the vulcanized specimen. Three tests were carried out at room temperature of 28 °C, and the average value was taken as the final test result. The thermal conductivity and specific heat capacity of the rubber material were 0.282 W/(m.K) and 1741 J/(kg.K), respectively.

### 2.4. Tensile Recovery Stress–Strain Curve Test

In this work, the uniaxial tension (UT) and biaxial tension (BT) tests were performed. The UT tests at the peak strain of 20%, 40%, 60%, 80%, and 100% were continuously performed with five cyclic stretches for each peak strain to eliminate the stress softening effect of the rubber. The BT tests at the peak strain of 20%, 40%, and 60% were performed with five cyclic stretches for each peak strain, following the same procedures as for in UT. Strain was measured with a laser extensometer.

### 2.5. Dynamic Properties Characterization of S−SBR

The quantitative relationship between S−SBR loss modulus and strain amplitude at different temperatures was tested using DMA+ 1000 dynamic mechanical analyzer by Metravib of France. The applied deformation was simple shear with a frequency of 10 Hz. The test swept the strain amplitude from a minimum strain amplitude of 0.1% to maximum of 100%. Temperature was scanned over the range from 25 °C to 105 °C in 10 °C intervals. The quantitative relationship between S−SBR loss modulus and strain rate was also measured using the DMA+ 1000 dynamic mechanical analyzer. The peak strain amplitude was fixed at 20%. The frequency was swept from 1 Hz to 50 Hz. Temperature was scanned over the range from 25 °C to 105 °C in 20 °C intervals.

### 2.6. HBU and RR Test of the Solid Tire

**Figure 2:**
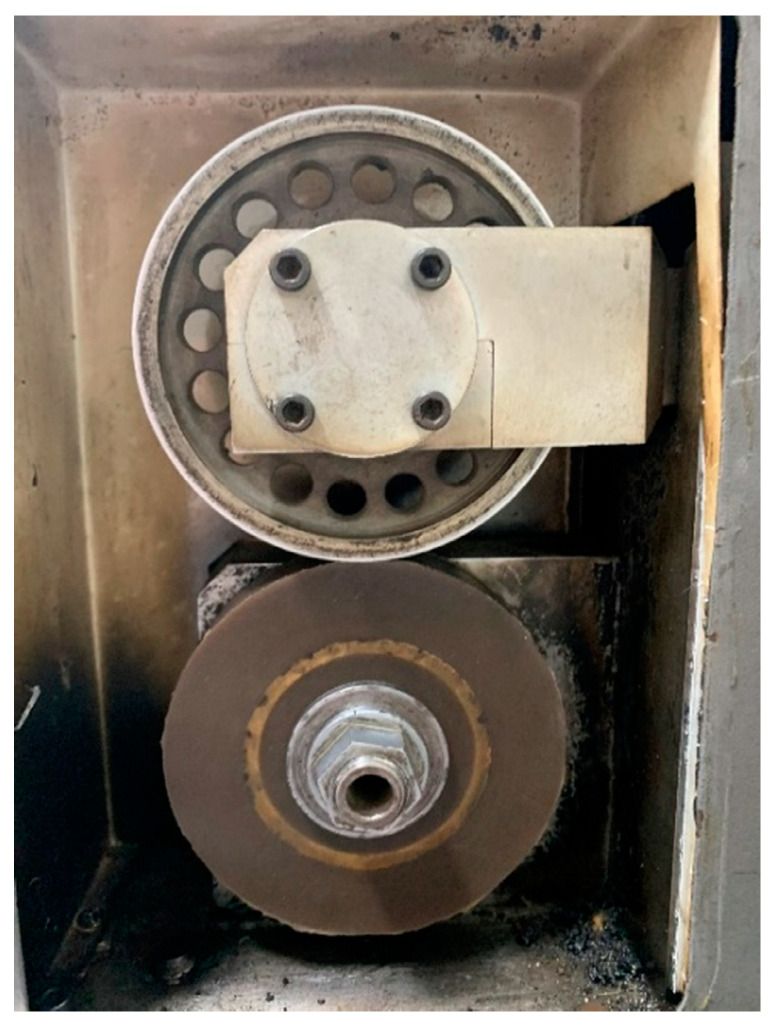
The RSS−II rolling resistance tester for solid rubber tires.

The HBU and RR of the prepared solid tire were measured at different rolling conditions by using the RSS−II rubber rolling resistance tester (Figure 2). The rolling resistance and surface temperature of the tire during the rolling process could be recorded. The boundary conditions are set as follows: (1) A load of 15 kg with a rotating speed of 600 rev/min; (2) a load of 30 kg with a rotating speed of 600 rev/min, and (3) a load of 30 kg with a rotating speed of 1200 rev/min. The duration time for each loading condition was 60 min. The infrared camera (FLIR A655sc) was used to record the surface temperature of the solid tire during rolling loading.

## 3. Flow Chart of Thermo-Mechanical Coupling Analysis of Solid Tires

**Figure 3:**
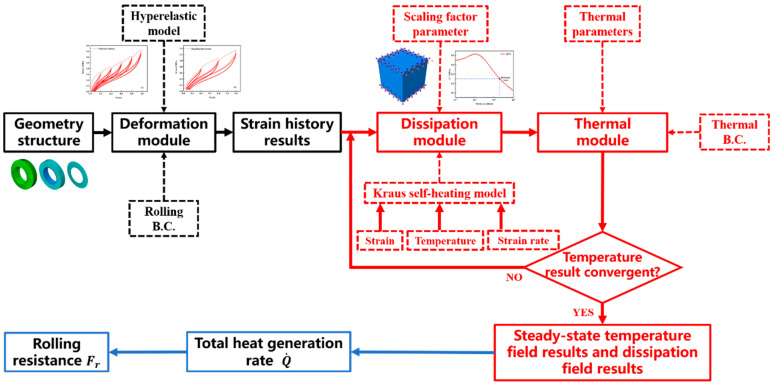
The flowchart of thermo-mechanical coupling analysis of the solid tire using Endurica and Abaqus co-simulation method.

The adopted flowchart of thermo-mechanical coupling analysis [17] of the solid tire is shown in Figure 3. The black part represents the deformation module, the red part represents the thermo-mechanical coupling module of Endurica and Abaqus co-simulation including the dissipation module and thermal module, and the blue part represents the RR calculation module. Combined with the determined hyperelastic constitutive model of S−SBR materials and the rolling boundary conditions (B.C.), we set up three different solid tire finite element models in the deformation module. The nominal strain component history results for each element in the solid tire model was obtained. In the co-simulation module, the heat generation rate of each element was calculated based on the Kraus self-heating model [17,18] and strain results computed in the deformation module. Due to the temperature, strain, and strain rate dependent dynamic properties of rubber, the HBU behavior is much more complicated [32,33,34]. Therefore, we need to introduce a co-simulation calculation method, that is, Endurica software for the calculation of the dissipation field of the solid tire model and Abaqus software for the calculation of the temperature field. The dissipation and temperature results need to be calculated iteratively until the temperature results converge. In the RR calculation module, we obtained the total heat generation rate $\dot{Q}$ of the whole solid tire model, and then we can calculate the rolling resistance *F_r_* of the tire model according to Equation (1). *v* is the rotating speed. In addition, the calculation time was recorded.

(1) $$F_{r}=\dot{Q}/v$$

## 4. RR and HBU Analysis of the Solid Tire

### 4.1. Three Finite Element Models of the Solid Tire under Straight-Ahead Rolling

1. Lagrangian–Eulerian finite element model

The Lagrangian–Eulerian finite element model is widely used to simulate steady state rolling of pneumatic tires. A standard Lagranian analysis (in which each node follows the motions of the material) is first made of the rim assembly, inflation, and static vertical loading steps. In the rolling analysis, an Eulerian method is used, (in which each node remains stationary in space while material flows through the mesh in correspondence with rotation of the tire).

**Figure 4:**
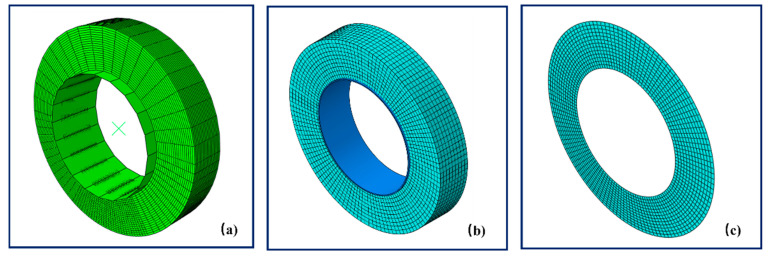
(**a**) The Lagrangian–Eulerian solid tire model, (**b**) the Lagrangian solid tire model, and (**c**) the Plane Strain solid tire model.

In the Lagrangian–Eulerian steady state rolling finite element model, the symmetric model generation (SMG), revolve, and symmetric results transfer (SRT) options in Abaqus were used to revolve the axisymmetric tire model into the full 3D tire model shown in Figure 4a. In the static and rolling analyses, the tire tread was attached to the hub via a tie constraint, and the road was represented by an analytic rigid surface. The total number of elements used in the 2D axisymmetric tire model was 90. 4-noded hybrid generalized bilinear axisymmetric quadrilateral (CGAX4H) elements were used. The total number of elements used in the 3D full tire model was 6660, consisting of 8-node hybrid linear brick (C3D8H) elements.

2. Lagrangian finite element solid rubber tire model

The biggest difference between the Lagrangian–Eulerian model and the Lagrangian model is that the latter tire model (shown in Figure 4b) no longer uses the Eulerian model in the rolling analysis, that is, the material and the mesh move together during rolling. The element type of the Lagrangian solid tire model is C3D8RH. The global seed was set to 2 mm, and total number of elements was 10,800. Strain history data were calculated for one rolling cycle of the tire.

3. Plane Strain finite element solid rubber tire model

The Plane Strain model is also a Lagrangian model shown in Figure 4c. Its computing time was reduced by reducing the number of elements in the model. The element type of the Plane Strain solid tire model is CPE4RH. The global seed was also set to 2 mm, and the total number of elements was 1638. Strain history data were calculated for one rolling cycle of the tire.

### 4.2. Hyperelastic Constitutive Model of S−SBR Material

**Figure 5:**
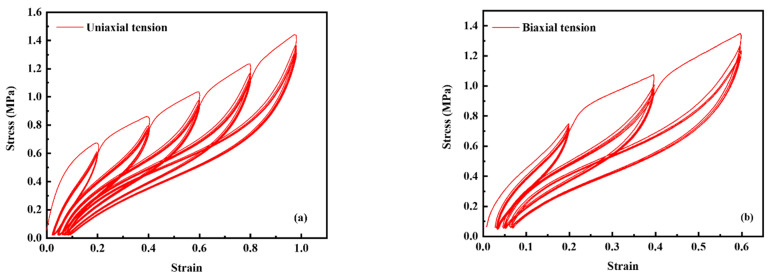
(**a**) The original cyclic stress–strain curves at five different peak strains in the deformation model of uniaxial tension, and (**b**) the original cyclic stress–strain curves at three different peak strains in the deformation model of biaxial tension.

**Figure 6:**
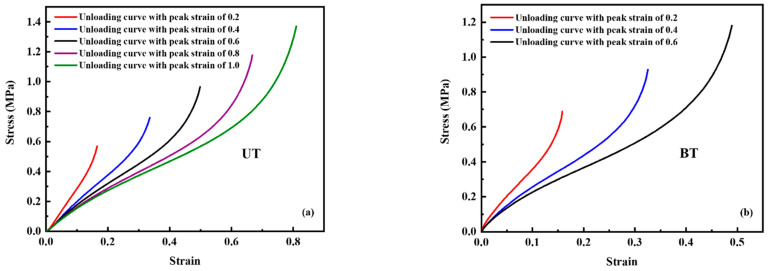
(**a**) The final adjusted unloading stress–strain curve at five peak strain levels of UT, and (**b**) the final adjusted unloading stress–strain curve at three peak strain levels of BT.

The tensile recovery stress strain curves in the deformation of UT and BT are shown in Figure 5. First, the last unloading curve at each peak strain level was extracted from the original loading-unloading stress–strain curves. Second, the permanent set (plastic deformation) can be eliminated for both UT and BT test data based on the Equations (2) and (3) reported in Mars’s work [35]. The adjusted stress–strain curve is shown in Figure 6.

(2) $$\varepsilon^{\prime }=\frac{1+\varepsilon }{1+\varepsilon_{set}}-1$$

(3) $$\sigma^{\prime }=\sigma_{0}(1+\varepsilon_{set})$$

In the Equations (2) and (3), *ε′* is the elastic strain, *ε* is the original nominal strain in the test data, *ε_set_* is the plastic strain, *σ′* is the elastic stress, *σ* is the original nominal stress in the test data. Third, we selected the final adjusted unloading stress–strain curve at five peak strain levels of UT and three peak strain levels of BT to fit the hyperelastic constitutive model. In other words, 15 different UT and BT combination test results can be used for hyperelastic model fitting.

**Figure 7:**
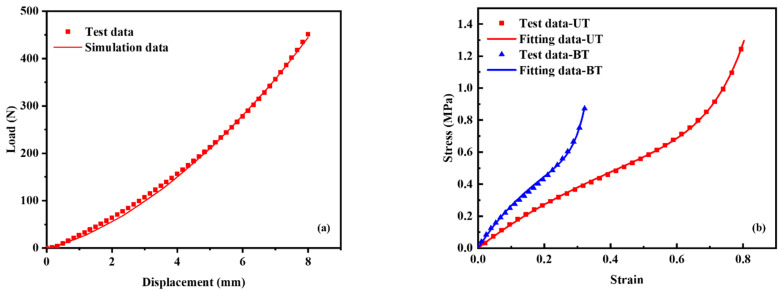
(**a**) The test data of reaction force vs. compressive displacement of the solid tire and the related simulation data obtained by the third-order Ogden hyperelastic model, and (**b**) the UT and BT test data and the related fitting data by the third-order Ogden hyperelastic model.

By comparing the experimental test data of the reaction force vs. compressive displacement with the simulation results of the solid tire shown in Figure 7a, it is found that the UT data with a peak strain of 100% and the BT data with a peak strain of 40% are the best input stress strain curves. A number of hyperelastic constitutive models can be used to describe the mechanical response of rubber materials [36]. The optimal hyperelastic constitutive model used to fit the test data is the third-order Ogden model shown in Figure 7b. The third-order Ogden model is shown in Equation (4). *W* is the strain energy density, *λ* is the stretch ratio. The subscripts 1, 2, and 3 stand for the three principal directions. Table 2 gives the material parameters of the Ogden model.

(4) $$W=\sum_{i=1}^{3}\frac{2\mu_{i}}{\alpha_{i}^{2}}({\bar{\lambda }}_{1}{}^{\alpha_{i}}+{\bar{\lambda }}_{2}{}^{\alpha_{i}}+{\bar{\lambda }}_{3}{}^{\alpha_{i}}-3)$$

### 4.3. Kraus Self-Heating Model

Accurate estimation of the hysteresis energy density is the key to a reliable simulation result of RR and HBU. The hysteresis energy density of rubber materials is closely related to temperature, strain amplitude, and strain rate. In order to obtain accurate simulation results, it is necessary to specify the nonlinear relationship between them [37]. In this work, we used the Kraus self-heating model [18] to establish the numerical relationship between the hysteresis energy density *h*, loss modulus *G’’*, strain amplitude *ε_a_*, temperature *θ*, and strain rate $\dot{\varepsilon }$ of the silica filled S−SBR material. The Kraus self-heating model is shown by Equation (5).

(5) $$h=CG^{\prime \prime }\varepsilon_{a}^{2}e^{r(\theta -\theta_{0})+Z(\dot{\varepsilon }-{\dot{\varepsilon }}_{0})}$$

In the formula, *C* is a scaling factor, *r* is temperature coefficient, *θ*_0_ is reference temperature, ${\dot{\varepsilon }}_{0}$ is reference strain rate, and *Z* is strain rate coefficient.

**Figure 8:**
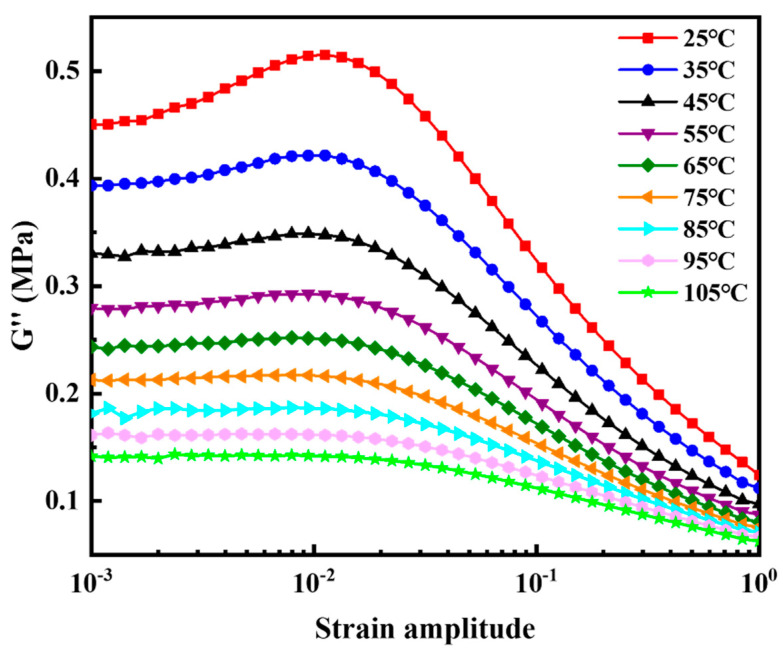
The dependence of loss modulus on strain amplitude at different temperatures. The strain sweeps from a small strain of 0.1% to a big one of 100%. Temperature scans from 25 °C to 105 °C in 10 °C intervals.

**Figure 9:**
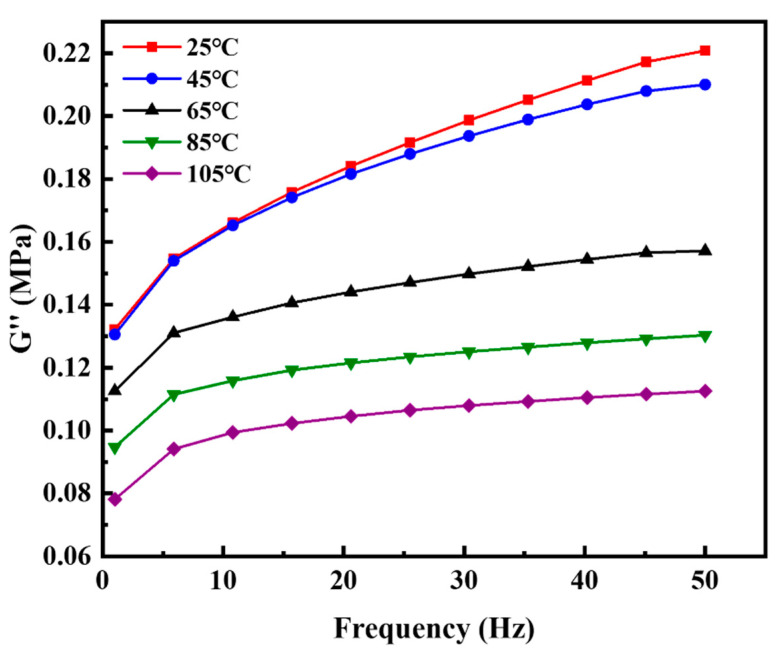
The dependence of loss modulus on frequency at different temperatures. The frequency sweeps from 1 Hz to 50 Hz. Temperature scans from 25 °C to 105 °C in 20 °C intervals.

The dynamic properties of the filled S−SBR material were systematically characterized. Figure 8 shows the dependence of loss modulus on strain amplitude at different temperatures. The strain amplitude swept from a minimum of 0.1% to a maximum of 100%. Temperature was scanned from 25 °C to 105 °C in 10 °C intervals. Figure 9 shows the dependence of loss modulus on frequency at different temperatures. The frequency swept from 1 Hz to 50 Hz. Temperature was scanned from 25 °C to 105 °C in 20 °C intervals.

#### 4.3.1. Parameter Fitting of the Kraus Self-Heating Model

1. The relation between *G’’* and *ε_a_* at a constant temperature and a constant frequency

**Figure 10:**
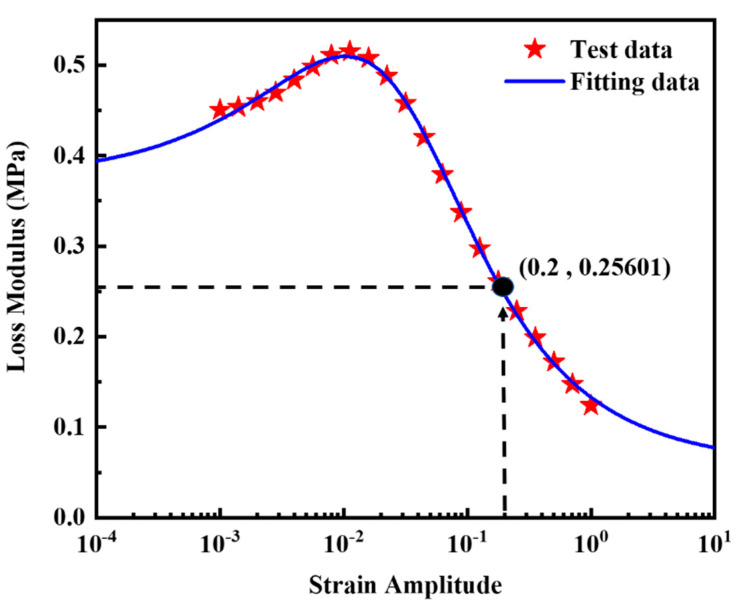
Test data of *G*″ vs. *ε_a_* and fitting data of the silica filled S−SBR material at a constant frequency of 10 Hz and a constant temperature of 25 °C. The coefficient of determination R^2^ is 0.9988.

Equation (6) shows the Kraus self-heating model including five material parameters $G_{\infty }^{\prime \prime }$, $G_{\max}^{\prime \prime }$, $\Delta G_{U}^{\prime \prime }$, *ε_a,c_* and *M* modified by Ulmer [38]. This model can very accurately describe the functional relation between *G’’* and *ε_a_* of rubber material [17,18]. The test data of loss modulus vs. strain amplitude at the temperature of 25 °C in Figure 8 was selected and fitted by Equation (6) at a constant frequency of 10 Hz and a constant temperature of 25 °C. Figure 10 shows Equation (6) can be used to accurately describe the relationship between *G*″ and *ε_a_*. The material parameter fitting results are given. $G_{\infty }^{\prime \prime }$, $G_{\max}^{\prime \prime }$, $\Delta G_{U}^{\prime \prime }$, *ε_a,c_* and *M* are 0.05729 MPa, 0.3054 MPa, 0.3166 MPa, 0.03029, and 0.5573, respectively.

(6) $$G^{\prime \prime }(\varepsilon_{a})=G_{\infty }^{\prime \prime }+\frac{(G_{\max}^{\prime \prime }-G_{\infty }^{\prime \prime }){\left(\varepsilon_{a}/\varepsilon_{a,c}\right)}^{M}+\Delta G_{U}^{\prime \prime }}{{\left(\varepsilon_{a}/\varepsilon_{a,c}\right)}^{2M}+1}$$

2. The relation between *h* and *θ* at a constant strain amplitude and a constant frequency

**Figure 11:**
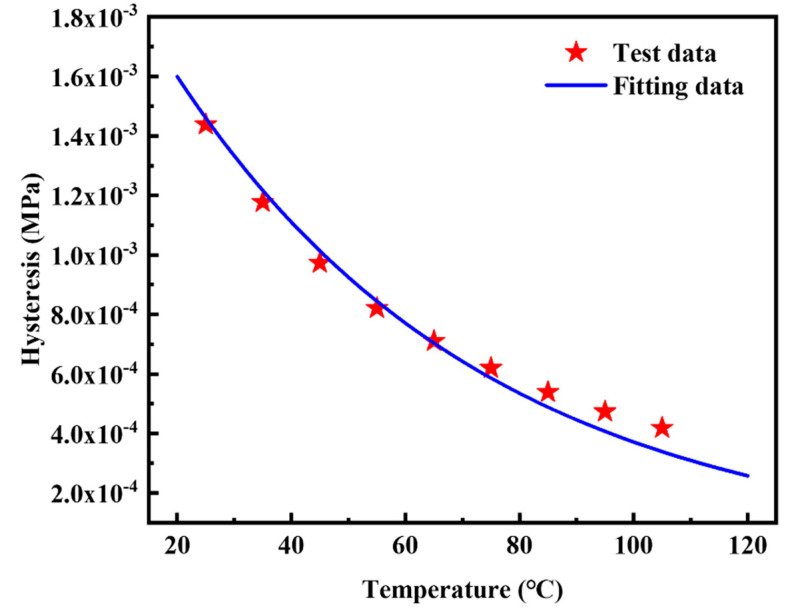
Test data of *h* vs. *θ* and fitting data of the silica filled S−SBR material at a constant strain amplitude of 20% and a constant frequency of 10 Hz. The coefficient of determination *R*^2^ is 0.9774.

The relationship between *G’’* and *ε_a_* has been constructed by the above Kraus self-heating model. In the thermo-mechanical coupling analysis, the quantitative relationship between *h* and *θ* is also necessary. Equation (7) was used to establish such a relation [17]. The reference temperature *θ*_0_ was set to 25 °C. The hysteresis energy density corresponding to the strain of 20% in Figure 8 was selected as the test data for fitting in Equation (7). The calculation relationship between the hysteresis energy density and the loss modulus is *h* = *πG*″*ε_a_*^2^. The basis for selecting the strain as 20% is because the maximum nominal strain of the contact area between tire and road is around 20%. Figure 11 indicates Equation (7) can be used to accurately describe the relationship between *h* and *θ*. The fitting results of the temperature coefficient *r* is −0.01825 1/°C.

(7) $$h(\theta )=a\cdot e^{r\cdot (\theta -\theta_{0})}$$

3 The relation between *h* and ${\dot{\varepsilon }}_{0}$ at a constant strain amplitude and a constant temperature

**Figure 12:**
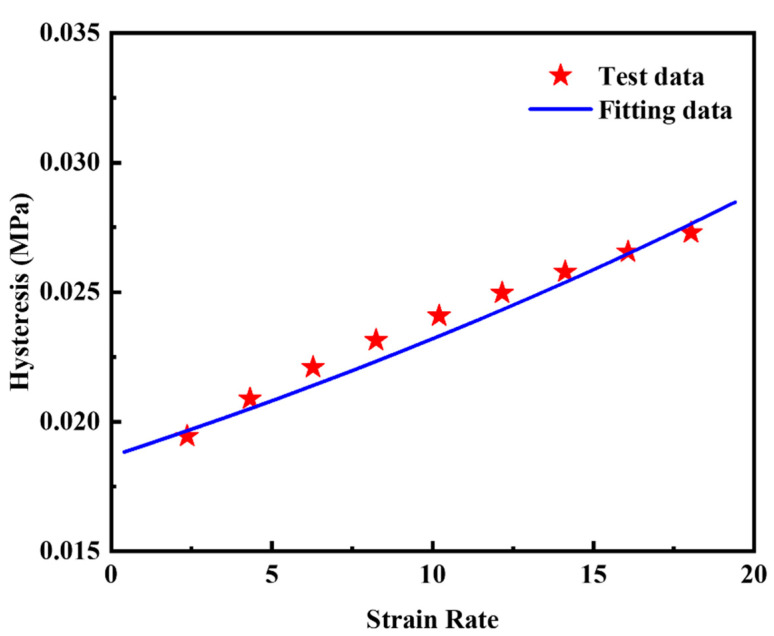
Test data of *h* vs. ${\dot{\varepsilon }}_{0}$ and fitting data of the silica filled S−SBR material at a constant strain amplitude of 0.4 and a constant temperature of 25 °C. The coefficient of determination *R^2^* is 0.9209.

The quantitative relationship between *h* and ${\dot{\varepsilon }}_{0}$ in the thermo-mechanical coupling analysis is established by Equation (9). Equation (8) gives the method for calculating the strain rate from the loading frequency. In the DMA test, the maximum strain *ε_max_* is 0.2 and the minimum strain *ε_min_* is −0.2. We choose the loading frequency of 10 Hz as the reference frequency, so the reference strain rate is 4 by Equation (8). The test data of loss modulus vs. frequency at the temperature of 25 °C in Figure 9 was selected and fitted by Equation (9) at a constant strain amplitude of 0.4 and a constant temperature of 25 °C. Figure 12 indicates Equation (9) can be used to describe the relationship between *h* and ${\dot{\varepsilon }}_{0}$. The fitting results of the strain rate coefficient *Z* is 0.02177 s.

(8) $${\dot{\varepsilon }}_{0}=(\varepsilon_{\max}-\varepsilon_{\min})\cdot f$$

(9) $$h(\dot{\varepsilon })=a\cdot e^{Z\cdot (\dot{\varepsilon }-{\dot{\varepsilon }}_{0})}$$

#### 4.3.2. Determination of the Scaling Factor

The scaling factor is the last key parameter in the Kraus self-heating model that must be determined. The purpose of introducing this parameter is to enforce agreement between the dissipated energy calculated by the microkinematic model with the experimental results. The scaling factor accounts for multiaxial loading, following the definition in Mars’s study on standing wave development, self-heating, and fatigue of pneumatic tire [17]. The scale factor was calculated based on the formula, *C* = ${\dot{Q}}_{test}$/${\dot{Q}}_{sim}$. ${\dot{Q}}_{test}$ is the test data of the energy dissipation rate at the reference temperature, reference strain amplitude, and reference strain rate. ${\dot{Q}}_{sim}$ is the simulation data of the energy dissipation rate (heat body flux per unit volume in Abaqus) at the same boundary conditions in the test. A simple shear simulation was conducted, and *C* was selected to match the simulated ${\dot{Q}}_{sim}$ with the experimental value ${\dot{Q}}_{test}$.

1. Determination of ${\dot{Q}}_{test}$

To determine ${\dot{Q}}_{test}$, the reference temperature, the reference strain amplitude and the reference frequency were set to 25 °C, 20%, and 10 Hz as shown by the arrow in Figure 10. ${\dot{Q}}_{test}$ can be calculated from the following formula: *π* × 0.25061 MPa ×0.2^2^ × 10 s^−1^ = 0.32171 mJ ⋅mm^−3^⋅s^−1^.

2. Determination of ${\dot{Q}}_{sim}$

**Figure 13:**
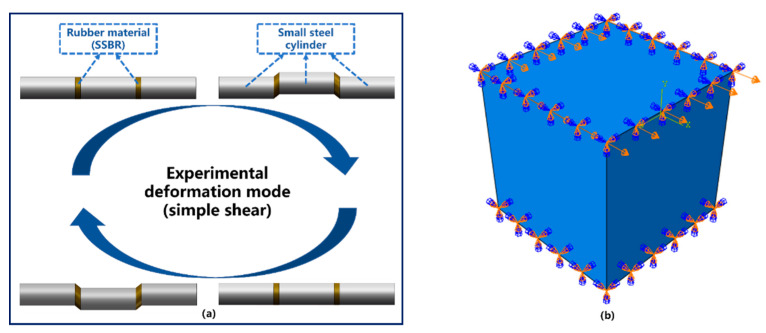
(**a**) The deformation mode of the specimen and the deformation of the material during the DMA test. (**b**) An eight-node element model for simulation, and its deformation modes.

A simple shear simulation was conducted to determine ${\dot{Q}}_{sim}$. To simplify the calculation, a unit cell with only one element and eight nodes was used as the finite element model shown in Figure 13b. The heat body flux per unit volume of the unit cell model can be computed using the co-simulation of Abaqus and Endurica. The initial value of the scaling factor was set to one. It is worth noting that the loading condition and deformation mode of the model in FEA were exactly the same as the test condition in DMA. In the simple shear test, the metal cylinders on both sides remain stationary, and the metal cylinder in the middle moves vertically with a strain amplitude of 20% shown in Figure 13a. Finally, the calculated ${\dot{Q}}_{sim}$ is 0.0117509 mJ ⋅mm^−3^⋅s^−1^.

3. Determination of the scaling factor *C*

The scaling factor is easily obtained, that is, *C* = ${\dot{Q}}_{test}$/${\dot{Q}}_{sim}$ = 27.4. We reset the scaling factor of the Kraus self-heating model to be 27.4 in the simulation work of the above unit cell, and the value of ${\dot{Q}}_{sim}$ is 0.317807 mJ ⋅mm^−3^⋅s^−1^. The relative error between ${\dot{Q}}_{test}$ and ${\dot{Q}}_{sim}$ is only 1.2%.

### 4.4. Boundary Conditions of Solid Tire

The structural boundary conditions in the three finite element models of the solid tires were exactly the same as the experimental test conditions. The boundary conditions were set as follows: (1) a load of 15 kg with a rotating speed of 600 rev/min, (2) a load of 30 kg with a rotating speed of 600 rev/min, and (3) a load of 30 kg with a rotating speed of 1200 rev/min. To simplify the calculation, the friction between the tire and the ground was set to frictionless.

The thermal boundary conditions were set as follows: For the 3D solid tire models with the rolling speed of 600 rev/min, the thermal convection coefficients for the tread and sidewall surfaces in direct contact with air were 0.032 mW/(mm^2^·K) and 0.024 mW/(mm^2^·K). In order to simplify the calculation, the inner side of the tire in contact with the metal rim is described by means of equivalent thermal convection. An equivalent thermal convection coefficient with the value of 0.140 mW/(mm^2^·K) was adopted to represent the heat transfer between them. When the rolling speed was 1200 rev/min, the thermal convection coefficients for the tread and sidewall surfaces were 0.044 mW/(mm^2^·K) and 0.037 mW/(mm^2^·K). The equivalent thermal convection coefficient for the inner side of the tire was still 0.140 mW/(mm^2^·K). Because the 2D model has no thickness in the lateral direction of the tire, that is to say, the thermal convection between the sidewall and the air cannot be considered. Compared with the 3D tire model, we only retained the thermal convection boundary conditions of the tread and inner side of the tire in contact with the metal rim, and the specific values remained unchanged.

## 5. Results and Discussion

### 5.1. HBU and RR Test Results of the Solid Tire at Different Loading Conditions

**Figure 14:**
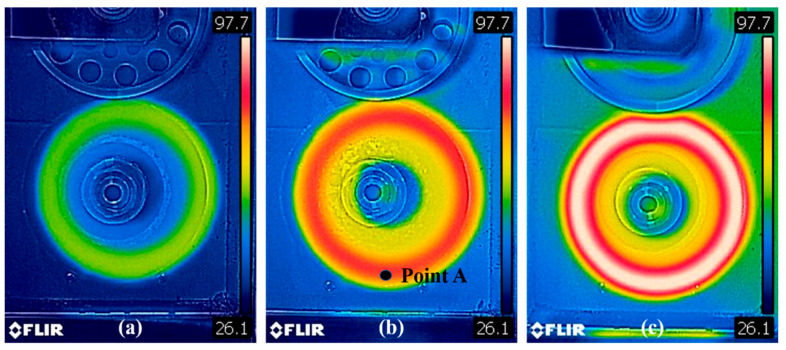
Steady-state surface temperature distribution of the solid tire recorded by an infrared camera under different boundary conditions (**a**) 15 kg and 600 rev/min, (**b**) 30 kg and 600 rev/min, (**c**) 30 kg and 1200 rev/min.

**Figure 15:**
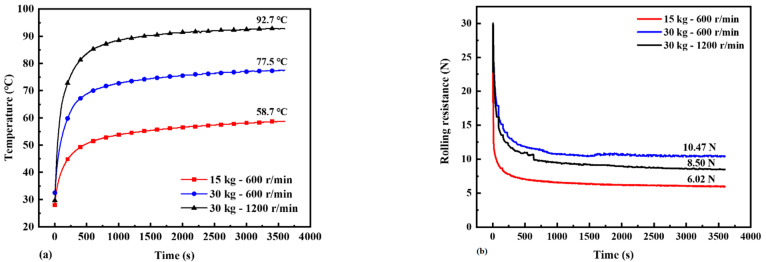
(**a**) Test data of the temperature vs. time curves at Point A shown in Figure 14b under different rotating speed and load, and (**b**) test data of the RR vs. time curves under different rotating speed and load.

The steady-state surface temperature distributions of the solid tire under different boundary conditions were recorded by an infrared camera shown in Figure 14. Figure 15 shows the test data of the transient surface temperature vs. time curves at Point A and RR vs. time curves of the solid tire under different rotating speed and load.

### 5.2. HBU and RR Simulation Results of the Three Solid Tire Finite Element Models at Different Loading Conditions

#### 5.2.1. Lagrangian–Eulerian Finite Element Model of the Solid Tire

**Figure 16:**
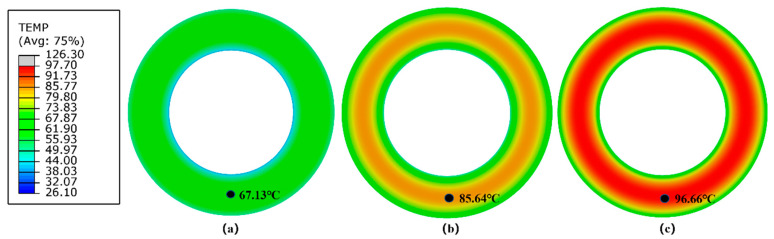
Contour plots of steady-state surface temperature of the Lagrangian–Eulerian finite element tire model under different rolling conditions (**a**) 15 kg and 600 rev/min, (**b**) 30 kg and 600 rev/min, (**c**) 30 kg and 1200 rev/min.

Contour plots of steady-state surface temperature of the Lagrangian–Eulerian tire model under three different rolling conditions are shown in Figure 16. Table 3 presents the numerical results of the RR and maximum steady-state surface temperature of the Lagrangian–Eulerian solid tire model under three rolling conditions.

#### 5.2.2. Lagrangian Finite Element Model of the Solid Tire

**Figure 17:**
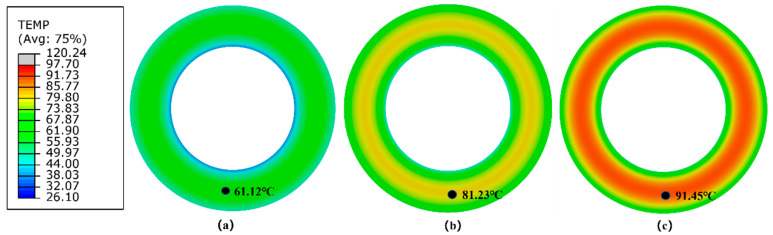
Contour plots of steady-state surface temperature of the Lagrangian finite element tire model under different rolling conditions (**a**) 15 kg and 600 rev/min, (**b**) 30 kg and 600 rev/min, (**c**) 30 kg and 1200 rev/min.

Contour plots of steady-state surface temperature of the Lagrangian tire model under three different rolling conditions are shown in Figure 17. Table 4 presents the numerical results of the RR and maximum steady-state surface temperature of the Lagrangian solid tire model under three rolling conditions.

#### 5.2.3. Plane Strain Finite Element Model of the Solid Tire

**Figure 18:**
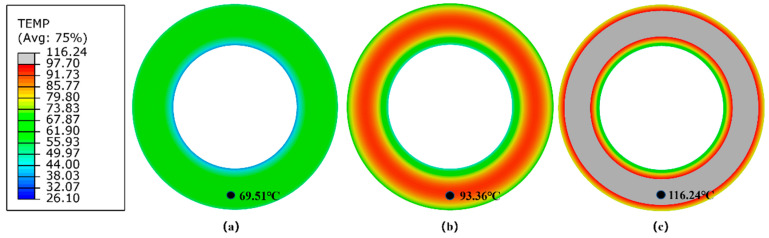
Contour plots of steady-state surface temperature of the Plane Strain finite element tire model under different rolling conditions (**a**) 15 kg and 600 rev/min, (**b**) 30 kg and 600 rev/min, (**c**) 30 kg and 1200 rev/min.

Contour plots of steady-state surface temperature of the Plane Strain tire model under three different rolling conditions are shown in Figure 18. Table 5 presents the numerical results of the RR and maximum steady-state surface temperature of the Plane Strain solid tire model under three rolling conditions.

### 5.3. Comparison between the Test Data and Simulation Data

By comparing the absolute values of test data and simulation data of the rolling resistance and the maximum steady-state surface temperature, we found that the simulation results and the test results are completely consistent in the changing trend. No matter which kind of finite element model of the solid tire is selected, the surface temperature of the solid tire increases with the increasing load while the rolling resistance decreases with the increasing rotating speed. This directly proves the effectiveness of the thermo-mechanical coupling analysis method established by the of co-simulation of Abaqus and Endurica in this work.

The relative error values of the simulation data and test data of the maximum steady-state surface temperature and rolling resistance of the three finite element models of the solid tire under different rolling conditions are summarized in Table 6. It can be seen from Table 6 that the minimum relative error is 1.4%, and the maximum relative error is no more than 50%. This quantitatively demonstrates the rationality of our finite element models, especially the material model and parameters, and the coupled thermo-mechanical analysis method.

Furthermore, we analyzed the differences of the three finite element models of the solid tire in predicting HBU and RR performance from the perspectives of calculation accuracy and calculation time. Overall, the Lagrangian model had the highest prediction accuracy, with an average prediction error of only 3.4% for maximum steady-state surface temperature and 7.9% for rolling resistance for the three rolling conditions. The Lagrangian–Eulerian model came in second, with an average prediction error of 9.7% for maximum steady-state surface temperature and 11.1% for rolling resistance. The prediction accuracy of the Plane Strain model was the worst, and the prediction errors of maximum steady-state surface temperature and rolling resistance were 21.4% and 44.6%, respectively.

However, when comparing the pros and cons of the three finite element models in terms of calculation time, we can easily find that this ordering was completely opposite to the ordering of prediction accuracy. The Plane Strain model had the lowest computational cost, with an average calculation time of 1143 s for the three rolling conditions. The Lagrangian–Eulerian model came in second, with an average calculation time of 2621 s. The calculation time of the Lagrangian model was the longest, 5597 s.

Through the systematic comparative analysis of the thermo-mechanical coupling analysis results of the above three finite element models and the experimental test results, we realize that the three finite element models of the solid tires have their own advantages and disadvantages. In the actual simulation requirements, we need to select the appropriate model according to the specific problem. For example, although the Plane Strain model has some shortcomings in prediction accuracy, its prediction trends for rolling resistance and heat build-up characteristics were in good agreement with the experimental results. When it is necessary to quickly understand the relationship between the performances of a rubber product and its structure and/or material properties, and the analytical object can be simplified to a two-dimensional plane problem, the plane stress or plane strain model is a good choice. For example, a lot of research work on non-pneumatic tires at Clemson University mentioned above is based on this type of model. The Lagrangian model shows advantages in terms of prediction accuracy. When the computational cost is not a major concern and high-precision analysis results are pursued, the Lagrangian model is recommended; for example, dynamic and static performance analysis of many rubber components such as bushing, bearings, hoses, and seals. The Lagrangian–Eulerian model takes into account both the prediction accuracy and the computational cost, and is currently widely used in the dynamic performance analysis of pneumatic tires.

## 6. Conclusions

1. A coupled thermo-mechanical approach was established to perform the maximum steady-state temperature and rolling resistance based on Endurica and Abaqus co-simulation. Special attentions were paid to the determination of material parameters in the hyperelastic model, the Kraus self-heating model and a key scaling factor of the silica filled S−SBR material. Systematic experimental tests were used to verify the effectiveness of simulation method.
2. The pros and cons of the three finite element models (Lagrangian–Eulerian model, Lagrangian model and Plane Strain model) of the solid tires under different boundary conditions were compared. The Lagrangian finite element model had the highest prediction accuracy and longest computational time. The Lagrangian–Eulerian finite element model came in the second place. The Plane Strain finite element model exhibited the best computational cost. The trend of the calculated results of the three models was completely consistent with the experimental results.
3. The validated thermo-mechanical coupling analysis method together with the three finite element models of the solid tires in this work can provide some reference for the optimization design of elastomeric components, pneumatic tires, and non-pneumatic tires in the future work.

## Figures and Tables

**Table 1:** Rubber formula of the green solid tire.

Name	Symbol	Amount (phr)
Solution styrene-butadiene rubber	S−SBR2550	160
Silica	VN3	75
Silane coupling agent	Si69	6
Zinc oxide	ZnO	2
Stearic acid	SA	3
Sulfur	S	1.5
Accelerator	CBS	1.5
Accelerator	D	2
Anti-aging agent	RD	1

**Table 2:** The material parameters in the third-order Ogden model.

*i*	$\mu_{i}$	$\alpha_{i}$
1	0.547376	1.720288
2	9.5996 × 10^−5^	19.50148
3	2.8502 ×10^−5^	−21.26955

**Table 3:** Simulation data of RR and maximum steady-state surface temperature of the Lagrangian–Eulerian model under different rolling conditions.

Boundary Condition	15 kg and 600 rev/min	30 kg and 600 rev/min	30 kg and 1200 rev/min
Rolling Resistance (N)	6.43	9.14	7.32
Surface Temperature (°C)	67.13	85.64	96.66

**Table 4:** Simulation data of RR and maximum steady-state surface temperature of the Lagrangian model under different rolling conditions.

Boundary Condition	15 kg and 600 rev/min	30 kg and 600 rev/min	30 kg and 1200 rev/min
Rolling Resistance (N)	5.76	9.58	7.58
Surface Temperature (°C)	61.12	81.23	91.45

**Table 5:** Simulation data of RR and maximum steady-state surface temperature of the Plane Strain model under different rolling conditions.

Boundary Condition	15 kg and 600 rev/min	30 kg and 600 rev/min	30 kg and 1200 rev/min
Rolling Resistance (N)	3.63	5.83	4.27
Surface Temperature (°C)	69.51	93.36	116.24

**Table 6:** The relative error values of the simulation data and test data of the maximum steady-state surface temperature and rolling resistance of the three finite element models of the solid tire under different rolling conditions, and the calculation time corresponding to all simulation analysis work.

Physical Quantity	Boundary Condition	Lagrangian -Eulerian	Lagrangian	Plane Strain
Maximum Surface Temperature (°C)	15 kg and 600 rev/min	14.4%	4.1%	18.4%
	30 kg and 600 rev/min	10.5%	4.8%	20.5%
	30 kg and 1200 rev/min	4.1%	1.4%	25.4%
Rolling Resistance(N)	15 kg and 600 rev/min	6.8%	4.3%	39.7%
	30 kg and 600 rev/min	12.7%	8.5%	44.3%
	30 kg and 1200 rev/min	13.9%	10.8%	49.8%
Calculation Time (s)	15 kg and 600 rev/min	1826	4275	801
	30 kg and 600 rev/min	2826	5301	1007
	30 kg and 1200 rev/min	3184	7215	1622
